# Importance of residency applicant factors based on specialty and demographics: a national survey of program directors

**DOI:** 10.1186/s12909-024-05267-8

**Published:** 2024-03-13

**Authors:** Sarah A. Strausser, Kelly M. Dopke, Destin Groff, Sue Boehmer, Robert P. Olympia

**Affiliations:** 1 Penn State College of Medicine, 17033 Hershey, PA USA; 2 Department of Public Health Services, Division of Biostatistics, Penn State Hershey Medical Center, Hershey, PA USA; 3 Department of Emergency Medicine, Penn State Milton S. Hershey Medical Center, 17033 Hershey, PA USA

**Keywords:** Medical education, Residency, Application

## Abstract

**Background:**

With the transition away from traditional numerical grades/scores, residency applicant factors such as service, research, leadership, and extra-curricular activities may become more critical in the application process.

**Objective:**

To assess the importance of residency application factors reported by program directors (PDs), stratified by director demographics and specialty.

**Method:**

A questionnaire was electronically distributed to 4241 residency PDs in 23 specialties during spring 2022 and included questions on PD demographics and 22 residency applicant factors, including demographics, academic history, research involvement, and extracurricular activities. Responses were measured using a Likert scale for importance. Descriptive statistics and Chi-square and Fisher exact test analysis were performed.

**Results:**

767 questionnaires were completed (19% response rate). Across all specialties, the factor considered most important was the interview (99.5%). When stratified by specialty, surgical PDs were more likely to characterize class rank, letters of recommendation, research, presenting scholarly work, and involvement in collegiate sports as extremely important/very important (all *p* < 0.0001). In contrast, primary care PDs favored the proximity of the candidate’s hometown (*p* = 0.0002) and community service (*p* = 0.03). Mean importance of applicant factors also differed by PD age, gender, and ethnicity.

**Conclusion:**

We have identified several residency application factors considered important by PDs, stratified by their specialty, demographics, and previous experiences. With the transition away from numerical grades/scores, medical students should be aware of the factors PDs consider important based on their chosen specialty. Our analysis may assist medical students in understanding the application and match process across various specialties.

**Supplementary Information:**

The online version contains supplementary material available at 10.1186/s12909-024-05267-8.

## Introduction

Several changes in medical school performance assessments have occurred over the past several years. The United States Medical Licensing Exam (USMLE) Step 1 transitioned from a numeric score to a pass/fail outcome on January 26, 2022 [1]. The score on this exam was previously considered one of the most important factors for choosing which residency applicants to interview, especially in applicants applying to competitive specialties [2]. Many medical school curriculums are graded as pass/fail during the preclinical years; thus, preclinical grades cannot be uniformly used to assess applicants. During the COVID-19 pandemic, numerous medical schools also changed their clinical year grading systems to pass/fail [3]. Given limited quantifiable data, these recent pass/fail reporting changes raise questions about how residency applicants should be objectively assessed.

The National Resident Matching Program (NRMP) Program Director Survey provides a wealth of information regarding factors that program directors value in selecting applicants to interview and ranking applicants in the Match. This survey is sent to residency program directors (PDs) of 23 specialties biennially, with the most recent report published in 2022 [4]. In 2021 and 2022, several items were deleted from the NRMP program director survey to allow space for more questions about holistic review and the virtual residency application experience during the pandemic, while decreasing respondents’ burden. Deleted items included questions about factors considered in decisions about which applicants to interview and rank and ratings of the importance of each factor. This survey compared the frequency with which programs interviewed and ranked specific applicant groups, including US MD Senior, US DO Senior, US MD Graduate, US DO Graduate, US IMG, and Non-US IMG. The survey also delved into the elements encompassed within the programs’ holistic review process and their significance. These factors include test scores, personal attributes, interests, interpersonal skills, ethics and professionalism, personal experiences, and geographic preferences.

While the NRMP survey results provide data regarding the mean importance of these holistic review factors, each of these factors is quite broad and does not characterize what specific attributes, interests, and experiences are of interest to program directors. In 2022, the NRMP stated that future iterations of the program director survey will re-introduce the questions about factors considered in decisions about which applicants to interview and rank. However, we felt it prudent to assess current opinions regarding which applicant factors are most important to program directors as programs continue to rely on a holistic review of applications due to decreasing objective data such as Step 1 scores.

Recent studies have surveyed program directors after the change to a pass/fail Step 1 score. One study found that approximately 40% of program directors replied that meaningful research participation would become more critical when choosing whom to offer interviews and that competitiveness of the specialty correlates with the reported importance of research [5]. Another study found that compared to non-procedural specialties, PDs of procedural specialties stated that they would place more emphasis on USMLE examinations such as Step 2 after transitioning to a pass/fail Step 1 [6]. While these studies, along with the NRMP data, provide substantial information regarding applicant factors that program directors emphasize when choosing whom to interview, the objective of our study was to analyze additional factors that may be emphasized in a holistic approach to applicant evaluation, such as athletic experience, military experience, medical scribe experience, and previous career prior to medicine. We evaluated the influence of athletic history on residency ranking given reported findings from other studies suggesting that prior participation in athletics may predict success in medical school and residency [7–9]. Medical scribe experience has also been postulated to be associated with academic success in medical school [10]. Military experience is associated with an increased likelihood of selecting a candidate for medical school interviews [11]. Secondarily, we assessed the importance of residency application factors stratified by PD demographics and specialty.

## Methods

Following Institutional Review Board approval at Penn State College of Medicine, a questionnaire was electronically distributed to 4241 residency PDs within 23 specialties during Spring 2022. PD contact information was collected for every specialty through the FREIDA (Fellowship and Residency Electronic Interactive Database Access) website. All PDs provided informed consent when they agreed to participate in the survey. The survey included seven questions on PD demographics and a list of 22 residency applicant factors with an associated Likert scale rating system where program directors would rank the importance of each factor, as shown in Appendix 1. This survey was developed by the authors, with no collection of validity evidence. Three reminder emails were sent at one-week intervals. Responses were stratified by specialty [surgical (general surgery, neurological surgery, obstetrics and gynecology, orthopaedic surgery, urology, otolaryngology, plastic surgery, and vascular surgery), primary care (family medicine, internal medicine, pediatrics, medicine-pediatrics), all other (anesthesiology, child neurology, dermatology, diagnostic radiology/nuclear medicine, emergency medicine, interventional radiology, neurology, pathology, physical medicine and rehabilitation, radiation oncology, and psychiatry)] and demographics of the PD (age, sex, ethnicity, previous extra-curricular experiences). Extremes on each end of the Likert scale were combined for analyses as the differences between “not at all important” and “slightly important” are unlikely to affect decision-making processes significantly. The same applies to the difference between “very important” and “extremely important.”

Percentages and 95% Confidence Intervals were calculated for categorical variables. Differences between groups for categorical variables were characterized using contingency table analysis; significance levels were determined by Pearson’s chi-square statistic and Fisher’s Exact Test. P-values less than 0.05 were considered significant. The statistical analysis was performed using SAS software, version 9.4.

## Results

**Fig. 1:**
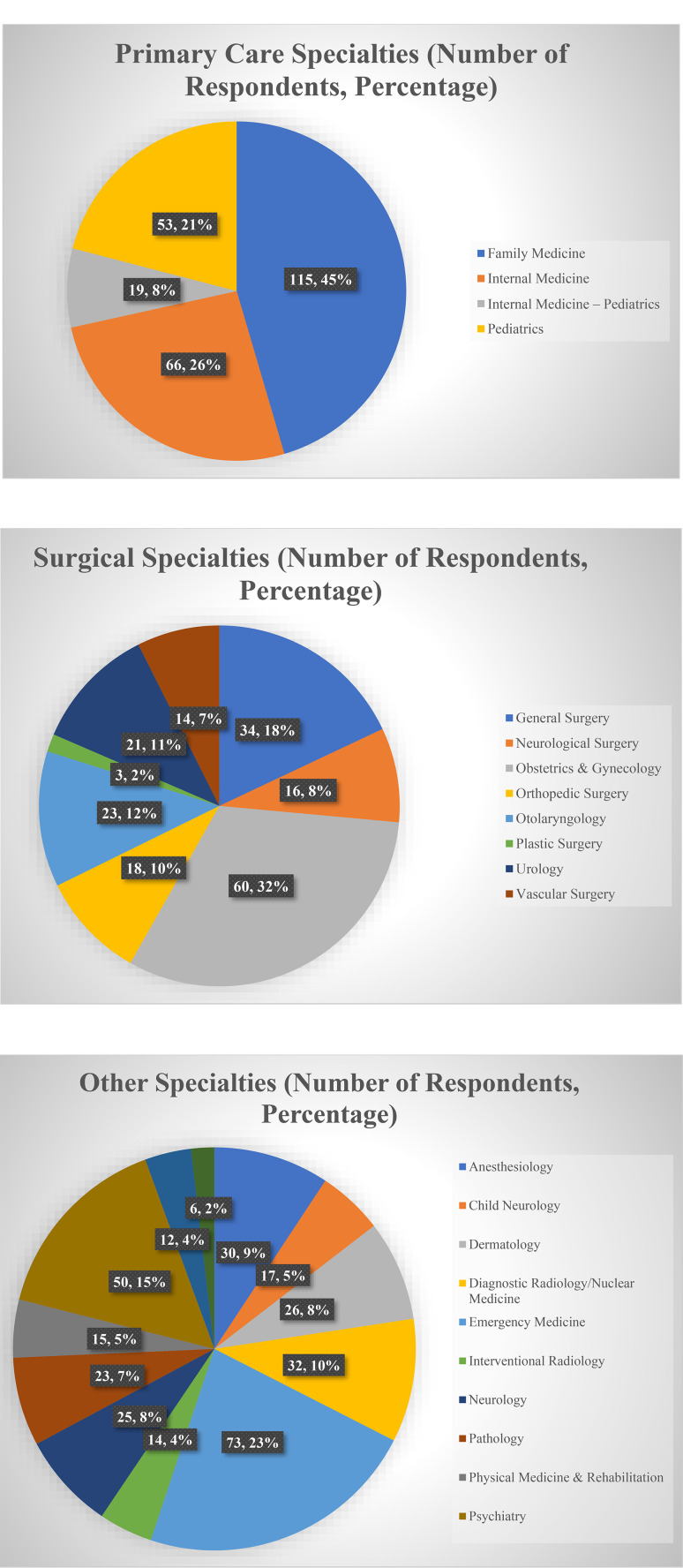
Respondents by specialty type (Primary Care, Surgical, Other)

Analysis was performed on 767 completed questionnaires (19% usable response rate). Most respondents were male (55.7%) and Caucasian (78.6%). 38.0% of respondents were between ages 41 and 50. (Table 1). Specialties of respondents included 189 surgical (25%), 253 primary care (33%), and 323 other (42%) (Fig. 1). The survey template is shown in Appendix 1. Across all specialties, the percentage of respondents who characterized the following factors as extremely important/very important were: interview (99.5%), passing USMLE examinations (88.2%), core clerkship grades (79.1%), demonstrating leadership (70%), letters of recommendation (69.4%), personal statement (64.2%), dean’s letter (49.4%), community service (40%), class rank (29.8%), specialty-specific research (19.3%), close proximity of candidate’s hometown (15.7%), research publications (15.1%), presenting their research at a scientific assembly (10.4%), ethnicity of candidate (9%), non-specialty-specific research (7.2%), previous involvement in collegiate sports (6.9%), previous career prior to medicine (5.5%), previous military experience (4.8%), previous involvement in global health (4.2%), sex of candidate (1.9%), previous involvement as a scribe (1.6%), and age of the applicant (1.4%) (Table 2). P-values less than 0.05 are bolded in Tables 2, 3, and 4 to distinguish significant findings.

**Table 1:** Demographics of responders (Percentage, 95% Confidence Interval)

		All Responders	PrimaryCare specialties	Surgical specialties	Other specialties	P value
Age	20–30 years	1/762 (0.1, 0.0-0.4)	0/251	0/188	1/322 (0.3,0.0-0.9)	0.28
	31–40 years	133/762 (17.5, 14.8–20.2)	42/251 (16.7,12.1–21.4)	26/188 (13.8,8.9–18.8)	65/322 (20.2,15.8–24.6)	
	41–50 years	288/762 (37.8, 34.4–41.2)	81/251 (32.3,26.5–38.1)	79/188 (42.0,34.9–49.1)	127/322 (39.4,34.1–44.8)	
	51–60 years	219/762 (28.7, 25.5–32.0)	80/251 (31.9,26.1–37.7)	53/188 (28.2,21.7–34.6)	86/322 (26.7,21.9–31.6)	
	61–70 years	102/762 (13.4, 11.0-15.8)	41/251 (16.3,11.8–20.9)	26/188 (13.8,8.9–18.8)	35/322 (10.9,7.5–14.3)	
	> 70 years	19/762 (2.5, 1.4–3.6)	7/251 (2.8,0.7–4.8)	4/188 (2.1,0.1–4.2)	8/322 (2.5,0.8–4.2)	
Gender	Male	420/754 (55.7, 52.2–59.3)	124/248 (50.0,43.8–56.2)	118/185 (63.8,56.8–70.7)	177/320 (55.3,49.9–60.)	0.02
	Female	331/754 (43.9, 40.4–47.4)	123/248 (49.6,43.4–55.8)	65/185 (35.1,28.2–42.0)	143/320 (44.7,39.2–50.1)	
	Other	3/754 (0.4, 0.0-0.9)	1/248 (0.4,0.0-1.2)	2/185 (1.1,0.0-2.6)	0/320	
Race	American Indian/Alaskan Native	5/767 (0.7, 0.1–1.2)	4/253 (1.6,0.4–3.1)	0/189	1/323 (0.3,0.0-0.9)	0.07
	Asian	73/767 (9.5, 7.4–11.6)	20/253 (7.9,4.6–11.2)	16/189 (8.5,4.5–12.4)	37/323 (11.5,8.0-14.9)	0.30
	Black/African-American	27/767 (3.5, 2.2–4.8)	11/253 (4.4,1.8–6.9)	4/189 (2.1,0.6–4.2)	12/323 (3.7,1.6–5.8)	0.44
	Hispanic/Latino	32/767 (4.2, 2.8–5.8)	11/253 (4.4,1.8–6.9)	7/189 (3.7,1.0-6.4)	14/323 (4.3,2.1–6.6)	0.93
	Native Hawaiian/Pacific Islander	4/767 (0.5, 0.0–1.0)	2/253 (0.8,0.0-1.9)	1/189 (0.5, 0.0-1.6)	1/323 (0.3,0.0-0.9)	0.73
	White/Caucasian	589/767 (76.8, 73.8–79.7)	193/253 (76.3,71.0-81.5)	149/189 (78.8,73.0-84.7)	247/323 (76.5,71.8–81.1)	0.79
	Other	16/767 (2.1, 1.1–3.1)				
	Prefer not to disclose	32/767 (4.2, 2.8–5.8)	11/253 (4.4,1.8–6.9)	7/189 (3.7,1.0-6.4)	14/323 (4.3,2.1–6.6)	0.93
How many years in clinical practice	0–5 years	28/763 (3.7, 2.3-5.0)	3/253 (1.2,0.0-2.5)	8/189 (4.2,1.4–7.1)	5/323 (1.6,0.2–2.9)	0.06
	0–5 years	28/763 (3.7, 2.3-5.0)	6/252 (2.4,0.5–4.3)	4/188 (2.1,0.1–4.2)	18/322 (5.6,3.1–8.1)	0.006
	6–10 years	135/763 (17.7, 15.0-20.4)	34/252 (13.5,9.3–17.7)	38/188 (20.2,14.5–26.0)	63/322 (19.6,15.2–23.9)	
	11–15 years	169/763 (22.2, 19.2–25.1)	47/252 (18.7,13.8–23.5)	44/188 (23.4,17.3–29.5)	78/322 (24.2,19.5–28.9)	
	16–20 years	126/763 (16.5, 13.9–19.2)	39/252 (15.5,11.0–20.0)	34/188 (18.1,12.6–23.6)	52/322 (16.2,12.1–20.2)	
	> 20 years	305/763 (40.0, 36.5–43.5)	126/252 (50.0,43.8–56.2)	68/188 (36.2,29.3–43.1)	111/322 (34.5,29.3–39.7)	
How many years as a residency director	< 1 year	35/762 (4.6, 3.1–6.1)	12/252 (4.8,2.1–7.4)	7/189 (3.7,1.0-6.4)	16/321 (5.0, 2.6–7.4)	0.74
	1–5 years	347/762 (45.5, 42.0-49.1)	105/252 (41.7,35.6–47.8)	88/189 (46.6,39.4–53.7)	154/321 (48.0,42.5-53.55)	
	6–10 years	206/762 (27.0, 23.9–30.2)	69/252 (27.4,21.9–32.9)	52/189 (27.5,21.1–33.9)	85/321 (26.5,21.6–31.3)	
	11–15 years	84/762 (11.0, 8.8–13.3)	31/252 (12.3,8.2–16.4)	24/189 (12.7,7.9–17.5)	29/321 (9.0,5.9–12.2)	
	16–20 years	40/762 (5.3, 3.7–6.8)	14/252 (5.6,2.7–8.4)	7/189 (3.7,1.0-6.4)	19/321 (5.9,3.3–8.5)	
	> 20 years	50/762 (6.6, 4.8–8.3)	21/252 (8.3,4.9–11.8)	11/189 (5.8,2.5–9.2)	18/321 (5.6,3.1–8.1)	
Military experience		45/767 (5.9, 4.2–7.5)	15/253 (5.9,3.0-8.8)	12/189 (6.4,2.9–9.8)	18/323 (5.6,3.1–8.1)	0.94
Collegiate varsity sports experience		102/767 (13.3, 10.9–15.7)	35/253 (13.8,9.6–18.1)	32/189 (16.9,11.6–22.3)	35/323 (10.8,7.4–14.2)	0.14
Global Health experience		122/767 (15.9, 13.3–18.5)	53/253 (21.0,15.9–26.0)	33/189 (17.5,12.0-22.9)	36/323 (11.2,7.7–14.6)	0.005
Medical scribe experience		11/767 (1.4, 0.6–2.3)	2/253 (1.9,0.0-1.9)	3/189 (1.6,0.0-3.4)	6/323 (1.9,0.4–3.3)	0.55

**Table 2:** Importance of applicant factors based on all responders and then stratified by specialty (Percentage, 95% Confidence Interval)

		All responders	Primarycare specialties	Surgical specialties	Other specialties	P value
Age of Candidate	Ext/very important	10/710 (1.4,0.5–2.3)	5/237 (2.1,0.3–3.9)	3/169 (1.8,0–3.8)	2/302 (0.7,0–1.6)	0.33
	Not at all/slight important	700/710 (98.6,97.7,99.5)	232/237 (97.9,96.1–99.7)	166/169 (98.2,96.2–100)	300/302 (99.3,98.4–100)	
Sex of Candidate	Ext/very important	13/698 (1.9,0.9–2.9)	1/246 (0.4,0–1.2)	6/169 (3.6,0.8–6.3)	6/281 (2.1,0.4–3.8)	0.06
	Not at all/slight important	685/698 (98.1,97.1–99.1)	245/246 (99.6,98.8–100)	163/169 (96.4,93.7–99.2)	275/281 (97.9,96.2–99.6)	
Ethnicity of Candidate	Ext/very important	51/565 (9.0,6.7–11.4)	17/182 (9.3,5.1–13.6)	13/149 (8.7,4.2–13.3)	21/232 (9.1,5.3–12.8)	0.98
	Not at all/slight important	514/565 (91.0,88.6–93.3)	165/182 (90.7,86.4–94.9)	136/149 (91.3,86.7–95.8)	211/232 (90.9,87.2–94.7)	
Proximity of candidate’s hometown to your program	Ext/very important	82/522 (15.7,12.6–18.8)	42/167 (25.2,18.5–31.8)	15/158 (9.5,4.9–14.1)	25/197 (12.7,8.0–17.4)	**0.0002**
	Not at all/slight important	440/522 (84.3,81.2–87.4)	125/167 (74.8,68.2–81.5)	143/158 (90.5,85.9–95.1)	172/197 (87.3,82.6–92.0)	
Previous career prior to medicine	Ext/very important	32/582 (5.5,3.6–7.4)	8/193 (4.2,1.3–7.0)	11/144 (7.6,3.3–12.0)	13/243 (5.4,2.5–8.2)	0.38
	Not at all/slight important	550/582 (94.5,92.6–96.4)	185/193 (95.8,93.0–98.7)	133/144 (92.4,88.0–96.7)	230/243 (94.6,91.8–97.5)	
Class Rank	Ext/very important	136/456 (29.8,25.6–34.0)	26/159 (16.4,10.6–22.1)	45/106 (42.5,33.0–51.9)	64/190 (33.7,26.9–73.1)	**< 0.0001**
	Not at all/slight important	320/456 (70.2,66.0–74.4)	133/159 (83.6,77.9–89.4)	61/106 (57.5,48.1–67.0)	126/190 (66.3,59.6–73.1)	
Passing the USMLE	Ext/very important	239/271 (88.2,84.3–92.1)	91/106 (85.9,79.2–92.5))	49/58 (84.5,75.1–93.9)	98/106 (92.5,87.4–97.5)	0.21
	Not at all/slight important	32/271 (11.8,7.9–15.7)	15/106 (14.1,7.5–20.8)	9/58 (15.5,6.1–24.9)	8/106 (7.5,2.5–12.6)	
Grades on core clerkships	Ext/very important	261/330 (79.1,74.7–83.5)	79/108 (73.2,64.7–81.6)	71/85 (83.5,75.6–91.5)	110/136 (80.9,74.2–87.5)	0.17
	Not at all/slight important	69/330 (20.9,16.5–25.3)	29/108 (26.8,18.4–35.3)	14/85 (16.5,8.5–24.4)	26/136 (19.1,12.5–25.8)	
Dean’s letter	Ext/very important	192/389 (49.4,44.4–54.3)	63/135 (46.7,38.2–55.1)	47/102 (46.1,36.4–55.8)	82/152 (54.0,46.0–61.9)	0.35
	Not at all/slight important	197/389 (50.6,45.7–55.6)	72/135 (53.3,44.9–61.8)	55/102 (53.9,44.2–63.6)	70/152 (46.0,38.1–54.0)	
Letters of recommendations	Ext/very important	247/356 (69.4,64.6–74.2)	74/137(54.0,45.6–62.4)	65/80 (81.3,72.7–89.8)	108 /139 (77.7,70.7–84.7)	**< 0.0001**
	Not at all/slight important	109/356 (30.6,25.8–35.4)	63/137 (46.0,37.6–54.4)	15/80 (18.7,10.2–27.3)	31/139 (22.3,15.3–29.3)	
Personal statement	Ext/very important	251/391 (64.2,59.4–69.0)	88/140 (62.9,54.8–70.9)	55/86 (64.0,53.8–74.1)	107/164 (65.2,57.9–72.6)	0.91
	Not at all/slight important	140/391 (35.8,31.0–40.6)	52/140 (37.1,29.1–45.2)	31/86 (36.0,25.9–46.2)	57/164 (34.8,27.4–42.1)	
Interview	Ext/very important	206/207 (99.5,98.6–100.0)	79/79 (100.0)	55/55 (100.0)	71/72 (98.6,95.9–100)	0.39
	Not at all/slight important	1/207 (0.5,0–1.4)	0/79 (0)	0/55 (0)	1/72 (1.4,0–4.1)	
Involvement in research pertaining to their specialty	Ext/very important	94/487 (19.3,15.8–22.8)	8/201 (4.0,1.3–6.7)	45/108 (41.7,32.3–51.0)	41/177 (23.2,16.9–29.4)	**< 0.0001**
	Not at all/slight important	393/487 (80.7,77.2–84.2)	193/201 (96.0,93.3–98.7)	63/108 (58.3,49.0–67.7)	136/177 (76.8,70.6–83.1)	
Involvement in research not pertaining to their specialty	Ext/very important	40/559 (7.2,5.0–9.3)	4/219 (1.8,0.05–3.6)	17/109 (15.6,8.8–22.4)	19/229 (8.3,4.7–11.9)	**< 0.0001**
	Not at all/slight important	519/559 (92.8,90.7–95.0)	215/219 (98.2,96.4–99.9)	92/109 (84.4,77.6–91.2)	210/229 (91.7,88.1–95.3)	
Presenting their research at a regional or national scientific assembly	Ext/very important	57/548 (10.4,7.8–13.0)	8/212 (3.8,1.2–6.3)	28/115 (24.4,16.5–32.2)	21/219 (9.6,5.7–13.5)	**< 0.0001**
	Not at all/slight important	491/548 (89.6,87.0–92.2)	204/212 (96.2,93.7–98.8)	87/115 (75.6,67.8–83.5)	198/219 (90.4,86.5–94.3)	
Publishing their research in a peer reviewed journal	Ext/very important	84/557 (15.1,12.1–18.1)	10/215 (4.7,1.8–7.5)	41/116 (35.3,26.6–44.1)	33/224 (14.7,10.1–19.3)	**< 0.0001**
	Not at all/slight important	473/557 (84.9,81.9–87.9)	205/215 (95.3,92.5–98.2)	75/116 (64.7,55.9–73.4)	191/224 (85.3,80.6–89.9)	
Demonstrating leadership	Ext/very important	260/372 (70.0,65.2–74.6)	74/119 (62.2,53.4–70.9)	73/91 (80.2,72.0–88.4)	113/160 (70.6,63.5–77.7)	**0.02**
	Not at all/slight important	112/372 (30.0,25.4–34.8)	45/119 (37.8,29.1–46.6)	18/91 (19.8,11.6–28.0)	47/160 (29.4,22.3–36.5)	
Previous military experience	Ext/very important	30/623 (4.8,3.1–6.5)	5/215 (2.3,0.3–4.3)	10/157 (6.4,2.5–10.2)	15/249 (6.0,3.1–9.0_	0.10
	Not at all/slight important	593/623 (95.2,93.5–96.9)	210/215 (97.7,95.7–99.7)	147/157 (93.6,89.8–97.5)	234/249 (94.0,91.0–96.9)	
Previous involvement in collegiate sports	Ext/very important	43/627 (6.9,4.9–8.8)	7/219 (3.2,0.9–5.5)	23/141 (16.3,10.2–22.4)	13/265 (4.9,2.3–7.5)	**< 0.0001**
	Not at all/slight important	584/627 (93.1,91.2–95.1)	212/219 (96.8,94.5–99.1)	118/141 (83.7,77.6–89.8)	252/265 (95.1,92.5–97.7)	
Previous experience as a scribe	Ext/very important	11/702 (1.6,0.6–2.5)	4/238 (1.7,0.1–3.3)	4/178 (2.3,0.1–4.4)	3/284 (1.1,0–2.2)	0.60
	Not at all/slight important	691/702 (98.4,97.5–99.4)	234/238 (98.3,96.7–99.9)	174/178 (97.7,95.6–99.9)	281/284 (98.9,97.8–100)	
Previous involvement in global health	Ext/very important	27/648 (4.2,2.6–5.7)	10/223 (4.5,1.8–7.2)	9/157 (5.7,2.1–9.4)	8/266 (3.0,0.9–5.1)	0.38
	Not at all/slight important	621/648 (95.8,94.3–97.4)	213/223 (95.5,92.8–98.2)	148/157 (94.3,90.6–97.9)	258/266 (97.0,94.9–99.1)	
Community service involvement	Ext/very important	170/415 (41.0,36.2–45.7)	65/128 (50.8,42.1–59.5)	41/112 (36.6,27.6–45.6)	64/173 (37.0,29.8–44.2)	**0.03**
	Not at all/slight important	245/415 (59.0,54.3–63.8)	63/128 (49.2,40.5–57.9)	71/112 (63.4,54.4–72.4)	109/173 (63.0,55.8–70.2)	

When stratified by specialty, surgical PDs were more likely to characterize the following factors as extremely important/very important: class rank (*p* < 0.0001), letters of recommendation (*p* < 0.0001), specialty-specific research (*p* < 0.0001), non-specialty-specific research (*p* < 0.0001), presenting their research at a scientific assembly (*p* < 0.0001), and previous involvement in collegiate sports (*p* < 0.0001). Primary care PDs were more likely to characterize the following factors as extremely important/very important: close proximity to the candidate’s hometown (*p* = 0.0002) and community service (*p* = 0.03) (Table 2). Data regarding the importance of residency applicant factors stratified by each specialty grouped within “all others” is displayed in the Supplemental File without p-values due to small sample sizes.

When stratified by demographics, PDs ≤ 50 years old were more likely than PDs ≥ 50 years old to characterize ethnicity (*p* = 0.04) and letters of recommendation (*p* = 0.03) as extremely important/very important. Male PDs were more likely to characterize class rank (*p* = 0.0007) as extremely important/very important. In contrast, female PDs were more likely to characterize ethnicity (*p* = 0.008) and community service (*p* = 0.005) as extremely important/very important. Non-Caucasian PDs were more likely to characterize proximity of hometown (*p* = 0.007), dean’s letter (*p* = 0.0003), letters of recommendation (*p* = 0.0003), involvement in global health (*p* = 0.001), and specialty-specific research (*p* = 0.0006) as extremely important/very important. PDs with previous collegiate sports experience were more likely to characterize leadership (*p* = 0.004) and previous involvement in collegiate athletics (*p* = 0.003) as extremely important/very important compared with PDs without athletics experience. PDs with prior global health experience were more likely to characterize previous involvement in global health (*p* = 0.009) and community service involvement (*p* = 0.002) as extremely important/very important compared to PDs without global health experience (Tables 3 and 4).

**Table 3:** Importance of applicant factors based on age, sex, and ethnicity of the responders (Percentage, 95% Confidence Interval)

		Age ≤ 50 years	Age > 50 years	P value	Male	Female	P value	Caucasian	Non-Caucasian	P value
Ethnicity of Candidate	Ext/very important	33/291 (11.3,7.7–15.0)	17/269 (6.3,3.4–9.2)	**0.04**	21/325 (6.5,3.8–9.1)	30/229 (13.1,8.7–17.5)	**0.008**	38/422 (9.0,6.3–11.7)	13/143 (9.1,4.4–13.8)	0.98
	Not at all/slight important	258/291 (88.7,85.0–92.3)	252/269 (93.7,90.8–96.6)		304/325 (93.5,90.8–96.2)	199/229 (86.9,82.5–91.3)		384/422 (91.0,88.3–93.7)	130/143 (90.9,86.2–95.6)	
Proximity hometown to program	Ext/very important	38/270 (14.1,9.9–18.2)	44/249 (17.7,12.9–22.4)	0.26	48/289 (16.6,12.3–20.9)	31/220 (14.1,9.5–18.7)	0.44	53/398 (13.3,9.9–16,7)	29/124 (23.4,15.9–30.9	**0.007**
	Not at all/slight important	232/291 (85.9,81.8–90.1)	205/249 (82.3,77.6–87.1)		241/289 (83.4,79.1–87.7)	189/220 (85.9,81.3–90.5)		345/398 (86.7,83.3–90.0)	95/124 (76.6,69.1–84.1)	
Class Rank	Ext/very important	75/262 (28.6,23.1–34.1)	60/191 (31.4,24.8–38.0)	0.52	91/250 (36.4,30.4–42.4)	43/199 (21.6,15.9–27.3)	**0.0007**	97/340 (28.5,23.7–33.3)	39/116 (33.6,25.0–42.2)	0.30
	Not at all/slight important	187/262 (71.4,65.9–76.9)	131/191 (68.6,62.0–75.2)		159/250 (63.6,57.6–69.6)	156/199 (78.4,72.6–84.1)		243/340 (71.5,66.7–76.2)	77/116 (66.4,57.7–75.0)	
Dean’s letter	Ext/very important	111/220 (50.5,43.8–57.1)	79/166 (47.6,40.0–55.2)	0.58	103/231 (44.6,38.1–51.0)	86/152 (56.6,48.7–64.5)	**0.02**	126/285 (44.2,38.4–50.0)	66/104 (63.5,54.2–72.7)	**0.0008**
	Not at all/slight important	109/220 (49.5,42.9–56.2)	87/166 (52.4,44.8–60.0)		128/231 (55.4,49.0–61.8)	66/152 (43.4,35.5–51.3)		159/285 (55.8,50.0–61.6)	38/104 (36.5,27.2–45.8)	
Letters of recommendations	Ext/very important	146/197 (74.1,68.0–80.3)	100/157 (63.7,56.1–71.3)	**0.03**	131/194 (67.5,60.9–74.1)	115/158 (72.8,65.8–79.7)	0.28	173/269 (64.3,58.6–70.1)	74/87 (85.1,77.5–92.6)	**0.0003**
	Not at all/slight important	51/197 (25.9,19.7–32.0)	57/157 (36.3,28.7–43.9)		63/194 (32.5,25.8–39.1)	43/158 (27.2,20.2–34.2)		96/269 (35.7,29.9–41.4)	13/87 (14.9,7.4–22.5)	
Personal statement	Ext/very important	119/202 (58.9,52.1–65.7)	130/186 (69.9,63.3–76.5)	**0.02**	132/221 (59.7,53.2–66.2)	115/162 (71.0,64.0–78.0)	**0.02**	181/295 (61.4,55.8–66.9)	70/96 (72.9,64.0–81.8)	**0.04**
	Not at all/slight important	83/202 (41.1,34.3–47.9)	56/186 (30.1,23.5–36.7)		89/221 (40.3,33.8–46.8)	47/162 (29.0,22.0–36.0)		114/295 (38.6,33.1–44.2)	26/96 (27.1,18.2–36.0)	
Inv. in research pert. to specialty	Ext/very important	59/273 (21.6,16.7–26.5)	34/210 (16.2,11.2–21.2)	0.13	56/258 (21.7, 16.6–26.7)	35/218 (16.1,11.2–20.9)	0.12	61/380 (16.1,12.3–19.7)	33/107 (30.8,22.1–39.6)	**0.0006**
	Not at all/slight important	214/273 (78.4,73.5–83.3)	176/210 (83.8,78.8–88.8)		202/258 (78.3,73.2–83.3)	183/218 (83.9,79.1–88.8)		319/380 (83.9,80.2–87.6)	74/107 (69.2,60.4–77.9)	
Presenting research at a reg/nat scientific assembly	Ext/very important	34/305 (11.2,7.6–14.7)	23/240 (9.6,5.8–13.3)	0.55	34/299 (11.4,7.8–15.0)	23/237 (9.7,5.9–13.5)	0.53	35/416 (8.4,5.7–11.1)	22/132 (16.7,10.3–23.0)	**0.007**
	Not at all/slight important	271/305 (88.8,85.3–92.4)	217/240 (90.4,86.7–94.2)		265/299 (88.6,85.0–92.2)	214/237 (90.3,86.5–94.1)		381/416 (91.6,88.9–94.3)	110/132 (83.3,77.0–89.7)	
Publishing research in a peer-rev. journal	Ext/very important	44/315 (14.0,10.1–17.8)	40/239 (16.7,12.0-21.5)	0.37	52/295 (17.6,13.3–22.0)	31/249 (12.5,8.3–16.6)	0.09	56/425 (13.2,9.9–16.4)	28/132 (21.2,14.2–28.2)	**0.02**
	Not at all/slight important	271/315 (86.0,82.2–89.9)	199/239 (83.3,78.5–88.0)		243/295 (82.4,78.0–86.7)	218/249 (87.5,83.4–91.7)		369/425 (86.8,83.6–90.0)	104/132 (78.8,71.8–85.8)	
Previous inv. in collegiate sports	Ext/very important	23/348 (6.6,4.0–9.2)	20/274 (7.3,4.2–10.4)	0.74	26/342 (7.6,4.8–10.4)	17/273 (6.2,3.4–9.1)	0.51	38/468 (8.1,5.6–10.6)	5/159 (3.1,0.4–5.9)	**0.03**
	Not at all/slight important	325/348 (93.4,90.8–96.0)	254/274 (92.7,89.6–95.8)		316/342 (92.4,89.6–95.2)	256/273 (93.8,90.9–96.6)		430/468 (91.9,89.4–94.4)	154/159 (96.9,94.1–99.6)	
Previous inv. in global health	Ext/very important	13/359 (3.6,1.7–5.6)	14/285 (4.9,2.4–7.4)	0.42	15/358 (4.2,2.1–6.3)	12/275 (4.4,1.9–6.8)	0.91	13/483 (2.7,1.2–4.1)	14/165 (8.5,4.2–12.7)	**0.001**
	Not at all/slight important	346/359 (96.4,94.4–98.3)	271/285 (95.1,92.6–97.6)		343/358 (95.8,93.7–97.9)	263/275 (95.6,93.2–98.0)		470/483 (97.3,95.9–98.8)	151/165 (91.5,87.3–95.8)	
Comm. service inv.	Ext/very important	89/223 (39.9,33.5–46.4)	81/190 (42.6,2 = 33.5–46.4)	0.58	83/236 (35.2,29.1–41.3)	83/169 (49.1,41.5–56.7)	**0.005**	131/318 (41.2,35.8–46.6)	39/97 (40.2,30.4–50.0)	0.86
	Not at all/slight important	134/223 (60.1,53.6–66.5)	109/190 (57.4,53.6–66.5)		153/236 (64.8,58.7–70.9)	86/169 (51.9,43.3–58.5)		187/318 (58.8,53.4–64.2)	58/97 (59.8,50.0–64.2)	

**Table 4:** Importance of applicant factors based on responders’ previous experience with the military, collegiate varsity sports, and global health (Percentage, 95% Confidence Interval)

		Military experience	No military experience	P value	Sports experience	No sports experience	P value	Global health experience	No global health experience	P value
Dem. leadership	Ext/very important	15/22 (68.2,48.6–87.7)	245/350 (70.0,65.2–74.8)	0.86	42/48 (87.5,78.1–96.9)	218/324 (67.3,62.1–72.4)	**0.004**	51/65 (78.5,68.4–88.5)	209/307 (68.1,62.8–73.3)	0.10
	Not at all/slight important	7/22 (31.8,12.3–51.4)	105/350 (30.0,25.2–34.8)		6/48 (12.5,3.1–21.9)	106/324 (32.7,27.6–37.8)		14/65 (21.5,11.5–31.6)	98/307 (31.9,26.7–37.2)	
Previous military exp.	Ext/very important	2/30 (6.7,0–15.6)	28/593 (4.7,3.0–6.4)	0.63	4/76 (5.3,0.2–10.3)	26/547 (4.8,3–6.5)	0.85	7/104 (6.7,1.9–11.6)	23/519 (4.4,2.7–6.2)	0.32
	Not at all/slight important	28/30 (93.3,84.4–100)	565/593 (95.3,93.6–97.0)		72/76 (94.7,89.7–99.8)	521/547 (95.2,93.4–97)		97/104 (93.3,88.4–98.1)	496/519 (95.6,93.8–97.3)	
Previous inv. in collegiate sports	Ext/very important	3/33 (9.1,0–18.9)	40/594 (6.7,4.7–8.7)	0.60	12/70 (17.1,8.3–26)	31/557 (5.6,3.6–7.5)	**0.0003**	3/94 (3.2,0–6.8)	40/533 (7.5,5.3–9.7)	0.13
	Not at all/slight important	30/33 (90.9,81.1–100)	554/594 (93.3,91.2–95.3)		58/70 (82.9,74–91.7	526/557 (94.4,92.5–96.3)		91/94 (96.8,93.2–100)	493/533 (92.5,90.3–94.7)	
Previous exp. as a scribe	Ext/very important	0/43 (0)	11/659 (1.7,0.7–2.6)	0.39	3/91 (3.3,0–7)	8/611 (1.3,0.4–2.2)	0.15	5/112 (4.5,0.6–8.3)	6/590 (1.0,0.2–1.8)	**0.007**
	Not at all/slight important	43/43 (100)	648/659 (98.3,97.4–99.3)		88/91 (96.7,93–100)	603/611 (98.7,97.8–99.6)		107/112 (95.5,91.7–99.4)	584/590 (99.0,98.2–99.8)	
Previous inv. in global health	Ext/very important	0/39 (0)	27/609 (4.4,2.8–6.1)	0.18	6/79 (7.6,1.7–13.4)	21/569 (3.7,2.1–5.2)	0.10	9/100 (9.0,3.4–14.6)	18/548 (3.3,1.8–4.8)	**0.009**
	Not at all/slight important	39/39 (100)	582/609 (95.6,93.9–97.2)		73/79 (92.4,86.5–98.3)	548/569 (96.3,94.7–97.8)		91/100 (91.0,85.4–96.6)	530/548 (96.7,95.2–98.2)	
Comm. service inv.	Ext/very important	9/28 (32.1,14.8–49.5)	161/387 (41.6,36.7–46.5)	0.33	22/49 (44.9,30.9–58.9)	148/366 (40.4,35.4–45.5)	0.55	38/65 (58.5,46.4–70.5)	132/350 (37.7,32.6–42.8)	**0.002**
	Not at all/slight important	19/28 (67.9,50.5–85.2)	226/387 (58.4,53.5–63.3)		27/49 (55.1,41.1–69.1)	218/366 (59.6,54.5–64.6)		27/65 (41.5,29.5–53.6)	218/350 (62.3,57.2–67.4)	

## Discussion

This survey analysis discovered numerous specific residency application factors considered important by PDs, stratified by their specialty, demographics, and previous experience.

Although several survey questions were similar to questions from the 2022 NRMP Program Director survey, our survey explores several applicant factors and their mean importance ratings that were asked in the 2020 NRMP Program Director Survey but not included in the 2022 NRMP Program Director Survey after Step 1 transitioned to pass/fail scoring. First, our survey uniquely stratifies responses by respondent demographics, allowing us to investigate how program director characteristics influence residency applicant ranking. Second, our survey uniquely expands on several residency applicant factors and supplements the 2022 NRMP survey as these aforementioned factors were removed to decrease respondent burden and focus on virtual aspects of residency applications during the pandemic, such as shifting to virtual recruitment and interviewing. Third, our survey focused on the importance of specific applicant experiences in residency ranking, including military experience, involvement in collegiate sports, experience as a scribe, and involvement in global health. Finally, our survey expanded on the importance of applicant demographics in residency ranking including age of candidate, sex of candidate, ethnicity of candidate, and proximity of candidate’s hometown to your program.

Several factors from the 2020 NRMP Program Director Survey overlapped with factors included in our survey. Across all specialties, factors in the NRMP survey were assessed for their importance in ranking applicants. Overlapping factors from the 2020 survey included several components of the interview, such as interpersonal skills (percent citing factor, average rating on Likert Scale with 1 being not at all important, 5 being very important) (95%, 4.8), interactions with faculty (89%, 4.8), and interactions with housestaff (89%, 4.8), letters of recommendation (70%, 4.1), leadership qualities (60%, 4.3), Dean’s Letter (58%, 4.0), personal statement (54%, 3.8), other life experience (47%, 4.0), any failed attempt in USMLE (46%, 4.4), grades in required clerkships (42%, 4.0), volunteer/extracurricular experiences (35%, 3.9) and demonstrated involvement and interest in research (28%, 3.8) [2]. Our results revealed similar emphasis on the importance of the interview, letters of recommendation, leadership qualities, Dean’ Letter, and the personal statement. Notably, in our survey conducted after the transition of Step1 scoring to pass/fail, 88.2% of respondents characterized passing USMLE examinations as extremely important/very important compared to 46% of respondents from the 2020 NRMP Program Director survey who cited any failed attempt in USMLE as an important factor in ranking applicants with an average rating of 4.4 on a Likert Scale of 1–5. This suggests that the importance of passing Step 1 on the first attempt has increased in importance since the transition to pass/fail scoring.

Our results also complement the 2022 NRMP Program Director survey. Program directors cited the following broad categories as important in holistic review of applicants: applicant personal attributes (88%, 4.4) (percent citing factor, average rating on Likert Scale with 1 being not at all important, 5 being very important), applicant interests (85%, 4.1), applicant interpersonal skills, ethics, and professionalism (81%, 4.6), and applicant personal experiences (81%, 3.9) [4].

Understanding our survey results may allow medical students to be better equipped in their decision-making regarding prospective residency programs in the medical specialty of their choice, knowing both the program director’s demographics and the subspecialty to which they are applying.

The decision to make the USMLE Step 1 exam pass/fail will have implications for both medical students and residency programs during the application process [6]. With this transition, in addition to many schools adopting the pass/fail curriculum for the didactic and clerkship years, students may find it harder to determine their competitiveness in a given specialty when relying solely on subjective feedback [12, 13]. In addition, without a 3-digit-score cutoff or student decision-making not to apply to specific programs due to a particular 3-digit score, the increasing number of applications each program receives will directly conflict with the holistic application screening because of the burden placed on educators to review many more applications. This may lead to using other objective measures, such as the USMLE Step 2, to become the screening tool of choice [14].

Given the limited amount of objective data, such as USMLE test scores on residency applications, it is essential for students to understand a residency program director’s perspective in the residency application process due to this consequential change. Unsurprisingly, program directors across all specialties emphasized application components, including interviews, passing USMLE examinations, core clerkship grades, demonstrating leadership, and letters of recommendation.

Our results indicate that PDs for surgical specialties will emphasize class rank, letters of recommendation, research, and previous involvement in collegiate sports. This is consistent with other studies analyzing factors that have an implication in the residency rank process [6, 15–17]. Thus, prospective candidates may glean insight into trends in applicants at specific programs by understanding the demographics of the program director of that residency program. The differences in applicant preference based on the demographics of PDs, including their age, sex, ethnicity, and athletic history, suggest implicit biases may influence the decision-making process for residency applicants. However, we feel that these data should not be used to guide students towards or away from certain programs based solely on PD demographics, but rather students should be aware that both implicit/explicit bias may affect applicant ranking during the residency application process. Additional studies are warranted to elucidate this potential relationship further.

There were several limitations to this study. First, we obtained program directors’ emails via FREIDA and found that many emails for program directors were not current, as we received undeliverable emails and responses that some PDs had left their positions. Another limitation is that our survey focused on USMLE scores rather than Comprehensive Osteopathic Medical Licensing Examination (COMLEX) scores, which osteopathic medical schools use. Future studies may include more emphasis on the role that COMLEX scores will play in residency applicant ranking.

There are several additional survey questions that we did not include but would supplement the findings of this study. For example, collecting demographic information about the size and rank/competitive nature of programs would help applicants better understand how program size and reputation affect the perceived importance of applicant factors. Our survey asked about the importance of factors on a Likert scale of 1–5. However, future iterations of surveys could include assessing if the presence or absence of factors, such as failure of a Step 1 exam, are critical in ranking applicants (i.e., a program may not rank an applicant with any USMLE failures). Another limitation is the omission of a free response text box for PDs to include additional comments of important aspects of candidate selection that were not included in our survey. We suggest a free response section be included in future PD surveys.

Our response rate of 19% is comparable to the 18% response rate for the 2020 NRMP Program Director survey, but lower than the 33.1% response rate for the 2022 NRMP Program Director survey. Although the data and generalizability of the results may be limited by the low response rate and sampling bias of those who choose to participate in the survey, we hope that our analysis of the importance of residency application factors assists medical students in understanding the application and match process across various specialties. Furthermore, we hope our data provides insight into possible implicit biases across specialties and how that may be implicated in the match process. Future research should involve determining whether medical students who excel or participate in these identified factors lead to success during the application and match process.

## Conclusion

We have identified several residency application factors considered important by PDs, stratified by their specialty, demographics, and previous experiences. With the transition away from numerical grades/scores, medical students should be aware of the factors PDs consider important based on their chosen specialty. Our analysis may assist medical students in understanding the application and match process across various specialties.

### Electronic supplementary material

Below is the link to the electronic supplementary material.

Supplementary Material 1

Supplementary Material 2

## Data Availability

The datasets used and/or analyzed during the current study are available from the corresponding author on reasonable request.

## Declarations

### Previous presentations

AAMC Learn Serve Lead Conference, Seattle, November 2023.
