# Supplementary material for: Importance of residency applicant factors based on specialty and demographics: a national survey of program directors
Source: BMC Med Educ. 2024 Mar 13;24:275. doi: 10.1186/s12909-024-05267-8 (PMC10935928; doi:10.1186/s12909-024-05267-8)
Supplement: Supplementary file 1 — Supplementary Material 1 [file 12909_2024_5267_MOESM1_ESM.pdf]

Program Director Survey

Title of Project: Factors Influencing Residency Applicant Ranking

Principal Investigator: Robert P. Olympia, MD

Address: PO Box 850: Emergency Medicine - H043, Hershey, PA 17033-0850 US
Telephone Numbers: Weekdays: 8:00 a.m. to 5:00 p.m. 717-531-8955

You are being invited to volunteer to participate in a research study. Research studies include only people who voluntarily choose to take part. This summary explains key information about this research. You are urged to ask questions about anything that is unclear to you.

- **The purpose of this study is to investigate residency candidate attributes that affect their ranking during the residency match process.**
- **Participation in this study involves completing an online survey in which you will answer 7 demographic questions and rank the importance of 22 factors taken into account when ranking a potential resident candidate for your residency program on a Likert scale of 5. (1 - Not at all important. 2 - Slightly important. 3 - Moderately important. 4 - Very important. 5 - Extremely important). You may skip any question that you do not wish to answer.**
- **This is a one-time survey which should take no more than 5 minutes to complete.**
- **There is a risk of loss of confidentiality if your information or your identity is obtained by someone other than the investigators, but precautions will be taken to prevent this from happening. The confidentiality of your electronic data created by you or by the researchers will be maintained to the degree permitted by the technology used. Absolute confidentiality cannot be guaranteed.**
- **Program directors and residency applicants may gain further understanding of the factors that influence ranking of residency applicants. This may help medical students prepare to apply to the specialty of their choice.**

You have the right to ask any questions you may have about this research. If you have questions, complaints or concerns or believe you may have been harmed from participating in this research, you should contact Dr. Robert P. Olympia at 717-531-8955. If you have questions regarding your rights as a research subject or concerns regarding your privacy, you may contact the research protection advocate in the HMC Human Subjects Protection Office at 717-531-5687. You may call this number to discuss any problems, concerns or questions; get information or offer input.

You do not have to participate in this research. Taking part in the research study is voluntary. Your decision to participate or to decline the research will not result in any penalty or loss of benefits to which you are entitled.

Your completion of the questionnaire implies your voluntary consent to participate in the research.

1. How old are you?

- ◯ 20-30 years
- ◯ 31-40 years
- ◯ 41-50 years
- ◯ 51-60 years
- ◯ 61-70 years
- ◯ >70 years

2. What is your gender?

- ◯ Male
- ◯ Female
- ◯ Non-Binary
- ◯ Other

2a. If Other, please specify.

____

3. What is your race? (Check all that apply.)

- ☐ American Indian/Alaskan Native
- ☐ Asian
- ☐ Black/African American
- ☐ Hispanic/Latino
- ☐ Native Hawaiian/Pacific Islander
- ☐ White
- ☐ Prefer not to disclose
- ☐ Other

3a. If Other, please specify.

____

4. What is your specialty?

- ◯ Anesthesiology
- ◯ Child Neurology
- ◯ Dermatology
- ◯ Diagnostic Radiology/Nuclear Medicine
- ◯ Emergency Medicine
- ◯ Family Medicine
- ◯ General Surgery
- ◯ Internal Medicine
- ◯ Internal Medicine - Pediatrics
- ◯ Interventional Radiology
- ◯ Neurological Surgery
- ◯ Neurology
- ◯ Obstetrics and Gynecology
- ◯ Orthopedic Surgery
- ◯ Urology
- ◯ Otolaryngology
- ◯ Pathology
- ◯ Pediatrics
- ◯ Physical Medicine and Rehabilitation
- ◯ Plastic Surgery
- ◯ Psychiatry
- ◯ Radiation Oncology
- ◯ Vascular Surgery
- ◯ Other

4a. If Other, please specify.

____

5. How many years have you been in clinical practice?

- ◯ 0-5 years
- ◯ 6-10 years
- ◯ 11-15 years
- ◯ 16-20 years
- ◯ >20 years

6. How many years have you been a residency director?

- ◯ < 1 year
- ◯ 1-5 years
- ◯ 6-10 years
- ◯ 11-15 years
- ◯ 16-20 years
- ◯ >20 years

7. Which of the following experiences have you had during or prior to medical training? (Check all that apply.)

- ☐ Military
- ☐ Medical Scribe
- ☐ Collegiate Varsity Sports
- ☐ Global Health

8. How important are each of the following factors when ranking a potential resident candidate for your residency program?

	1 - Not at all Important	2 - Slightly Important	3 - Moderately Important	4 - Very Important	5 - Extremely Important
Age of candidate	◯	◯	◯	◯	◯
Sex of candidate	◯	◯	◯	◯	◯
Ethnicity of candidate	◯	◯	◯	◯	◯
Proximity of candidate's hometown to your program	◯	◯	◯	◯	◯
Previous career prior to medicine	◯	◯	◯	◯	◯

9. How important are each of the following factors when ranking a potential resident candidate for your residency program?

	1 - Not at all important	2 - Slightly important	3 - Moderately important	4 - Very important	5 - Extremely important
Class Rank	◯	◯	◯	◯	◯
Passing USMLE	◯	◯	◯	◯	◯
Grades on core clerkships	◯	◯	◯	◯	◯
Dean's letter	◯	◯	◯	◯	◯
Letters of recommendation	◯	◯	◯	◯	◯
Personal statement	◯	◯	◯	◯	◯
Interview	◯	◯	◯	◯	◯

10. How important are each of the following factors when ranking a potential resident candidate for your residency program?

	1 - Not at all Important	2 - Slightly Important	3 - Moderately Important	4 - Very Important	5 - Extremely Important
Involvement in research pertaining to your specialty	◯	◯	◯	◯	◯
Involvement in research not pertaining to your specialty	◯	◯	◯	◯	◯

Presenting their research at a regional or national scientific assembly	○	○	○	○	○
Publishing their research in a peer reviewed journal	○	○	○	○	○

11. How important are each of the following factors when ranking a potential resident candidate for your residency program?

	1 - Not at all Important	2 - Slightly Important	3 - Moderately Important	4 - Very Important	5 - Extremely Important
Demonstrating leadership during medical school	○	○	○	○	○
Previous military experience	○	○	○	○	○
Previous involvement in collegiate sports	○	○	○	○	○
Previous position as a scribe	○	○	○	○	○
Previous involvement in global health	○	○	○	○	○
Community service during medical school	○	○	○	○	○
